# Permafrost cooled in winter by thermal bridging through snow-covered shrub branches

**DOI:** 10.1038/s41561-022-00979-2

**Published:** 2022-07-07

**Authors:** Florent Domine, Kévin Fourteau, Ghislain Picard, Georg Lackner, Denis Sarrazin, Mathilde Poirier

**Affiliations:** 1 Takuvik Joint International Laboratory, Université Laval (Canada) and CNRS-INSU (France), Québec, Canada; 2 Centre d’Études Nordiques, Université Laval, Québec, Canada; 3 Department of Chemistry, Université Laval, Québec, Canada; 4 Université Grenoble Alpes, Université Toulouse, Météo-France, CNRS, CNRM, Centre d’Études de la Neige, Grenoble, France; 5 Université Grenoble Alpes, CNRS, IRD, Grenoble INP, IGE, Grenoble, France; 6 Department of Civil and Water Engineering, Université Laval, Québec, Canada; 7 Department of Biology, Université Laval, Québec, Canada

**Keywords:** Cryospheric science, Climate-change impacts, Climate change, Climate sciences

## Abstract

Considerable expansion of shrubs across the Arctic tundra has been observed in recent decades. These shrubs are thought to have a warming effect on permafrost by increasing snowpack thermal insulation, thereby limiting winter cooling and accelerating thaw. Here, we use ground temperature observations and heat transfer simulations to show that low shrubs can actually cool the ground in winter by providing a thermal bridge through the snowpack. Observations from unmanipulated herb tundra and shrub tundra sites on Bylot Island in the Canadian high Arctic reveal a 1.21 °C cooling effect between November and February. This is despite a snowpack that is twice as insulating in shrubs. The thermal bridging effect is reversed in spring when shrub branches absorb solar radiation and transfer heat to the ground. The overall thermal effect is likely to depend on snow and shrub characteristics and terrain aspect. The inclusion of these thermal bridging processes into climate models may have an important impact on projected greenhouse gas emissions by permafrost.

## Abstract (web summary)

Arctic shrubs cool permafrost in winter by acting as a thermal bridge through the snowpack, according to ground temperature observations and heat transfer simulations.

## Main

Permafrost stores about 1,400 Pg of frozen carbon, mostly in the form of decomposing vegetal material^1^. Permafrost thaw accelerates the metabolism of soil microbes, increasing the release of the greenhouse gases (GHG) CO_2_ and CH_4_ (ref. ^2^). The rate of thawing is affected by little-understood feedbacks^3^. Shrub expansion in the Arctic^4^ is suspected of accelerating permafrost thaw by increasing snow accumulation^5,6^, which reduces winter cooling by insulating permafrost from the cold winter air. Furthermore, shrubs enhance snowpack insulation in the high Arctic by favouring the formation of depth hoar, a highly insulating snow type, at the expense of more conductive wind slabs prevailing over wind-swept herb tundra^6,7^.

Several Arctic field observations and manipulations indicate that shrub expansion leads to permafrost winter warming. One study^6^ observed that in shrubs, the snow was 60% more insulating at shrub sites than at tussock tundra sites, resulting in 3 °C warmer soils. Dead shrubs placed on open tundra resulted in topsoil warming by 4–5 °C in January^8^. Another study^9^ found that March temperatures were about 2.5 °C and 5 °C warmer under dwarf shrubs and tall shrubs, respectively, than under lichen. In these three studies, snow at shrub sites was thicker than in the absence of shrubs.

**Fig. 1:**
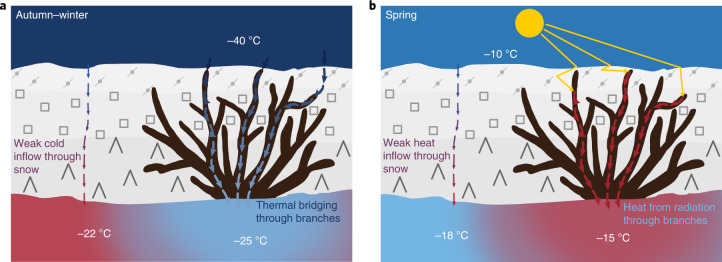
Thermal bridging through shrub branches in winter and spring. **a**, In autumn and winter, in the absence of sunlight, thermal bridging through frozen shrub branches cools the ground. **b**, In spring, light absorption by shallow buried shrub branches heats up the branches. The resulting heat is transferred through the branches to the ground, whose warming is thus accelerated.

At Bylot Island (73° N), previous studies^7^ reported a greater proportion of depth hoar in willow shrubs and measured mean snow thermal conductivities 29% lower in willows than on herb tundra. However, snow was not thicker in willows. Simulations of the permafrost thermal regime under willows and under herb tundra, accounting for snow differences, indicated that minimum winter permafrost temperature should be 7–13 °C warmer under willows. This large shrub-induced warming motivated the installation of instruments at shrub and tundra sites to test model predictions. Three years of monitoring contradict predictions and show that shrubs lead to ground cooling in winter. Here, we propose a new process, currently not considered in permafrost studies^10^, to explain observations. We propose that at Bylot Island, frozen shrub branches that extend to or near the snowpack surface, and which conduct heat about 20 times better than snow (see Methods), act as highly conducting thermal bridges that enhance permafrost cooling in winter, overriding the effect of the more insulating snowpack. In spring, a second process is detected: buried branches absorb solar radiation under the snow and conduct heat to the ground, accelerating ground spring warming (Fig. 1). In current models, the omission of conduction through, and radiation absorption by, branches underestimates cooling in winter and underestimates warming in spring. The reality of the permafrost thermal regime under shrubs is not captured, with consequence on permafrost temperature^10^, nutrient recycling^11–13^, plant development^14^, GHG winter and spring emissions^15–17^, and geomorphological changes^18^, resulting in the inaccurate quantification of the shrub expansion–permafrost–climate feedback^6^.

## Observed winter cooling

**Extended Data Fig. 1:**
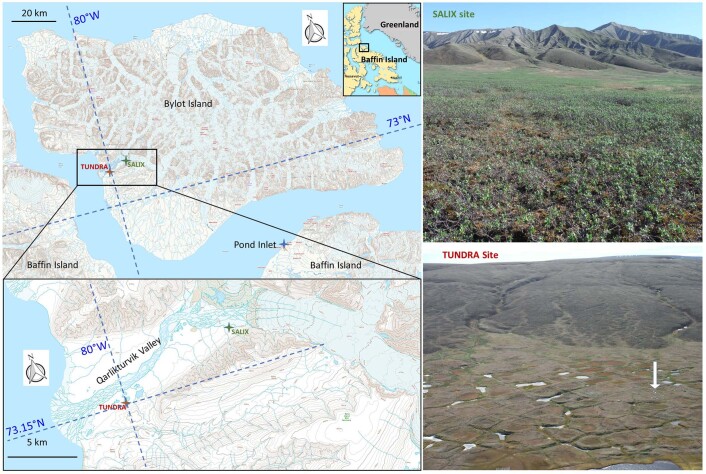
Map and photographs of study sites. Qarlikturvik valley is on Bylot Island, Canada (73°N, 80°W). Top right: photographs of the SALIX site (73.1816°N, 79.7454°W, 35 m elevation) before instrument installation. Bottom right: aerial photograph of the TUNDRA site (73.1504°N, 80.0046°W, 15 m elevation). Instruments location at the TUNDRA site is indicated by the white arrow. Maps from atlas.gc.ca last accessed on 25 April 2022.

We worked in Qarlikturvik valley, Bylot Island, north of Baffin Island in the Canadian high Arctic (Extended Data Fig. 1 and Supplementary Fig. 1). We selected a herb tundra site (TUNDRA) and a shrub site (SALIX) with 35–40-cm-high willows (*Salix richardsonii*), 9 km upvalley from TUNDRA. TUNDRA is near the middle of a low-centre polygon. The general topography has a slope of about 1° with north–northwesterly aspect. SALIX, on a slope of about 3° with northerly aspect, is downhill of an alluvial fan and a few metres from flow channels active during melt and heavy rains.

**Fig. 2:**
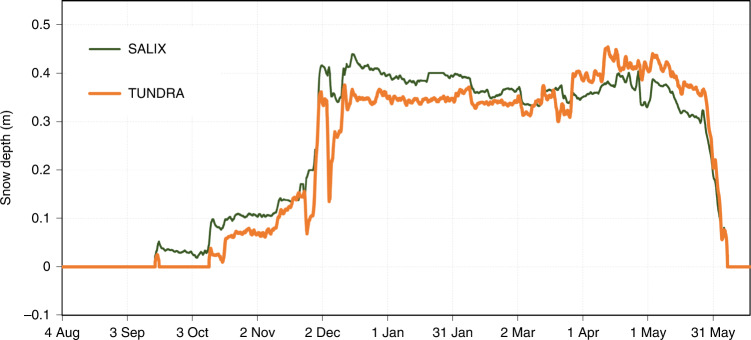
Snow depth at shrub and herb tundra sites during 2018–2019. Observed snow depth at the shrub site (SALIX) and the herb tundra site (TUNDRA) in the Canadian high Arctic.

**Fig. 3:**
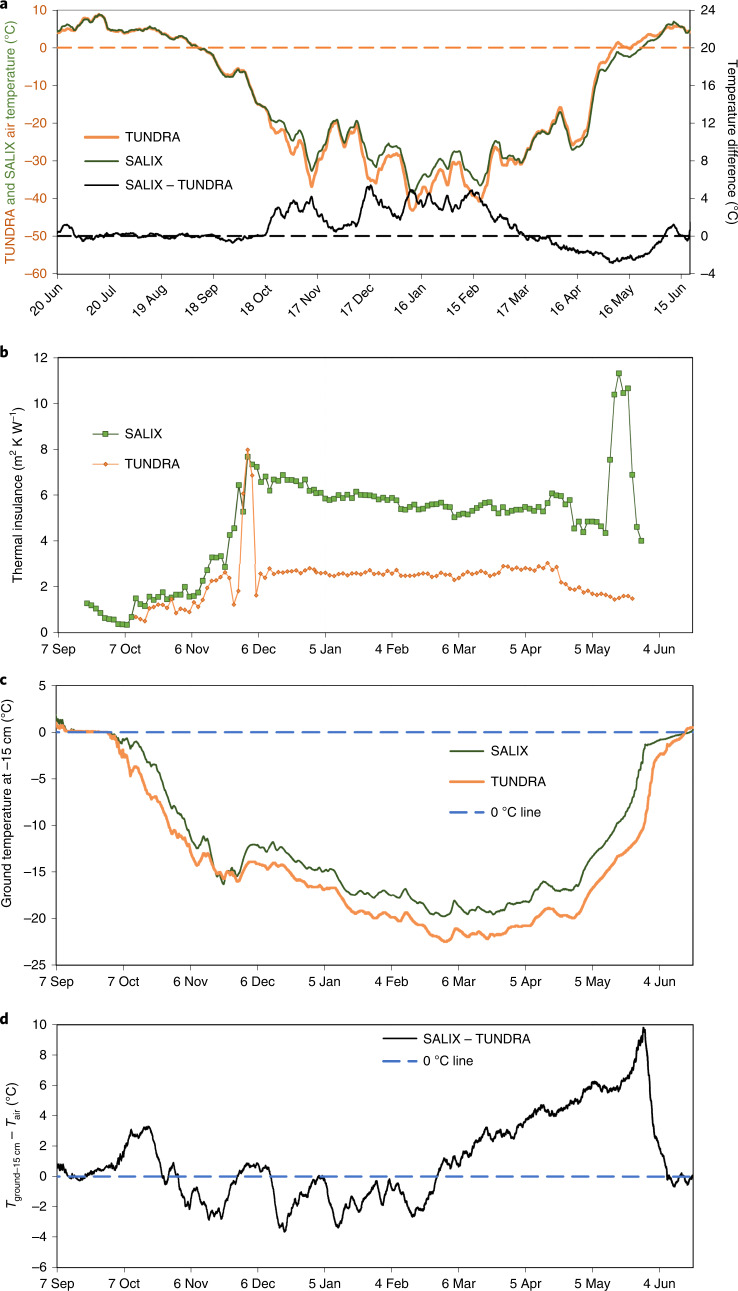
Thermal variables at SALIX and TUNDRA for 2018–2019. **a**, One-week running mean air temperature and one-week running mean temperature difference between both sites. **b**, Snowpack thermal insulance. **c**, One-week running mean temperature of the ground at 15 cm depth at both sites. These data have not been corrected for air temperature differences between both sites. **d**, Temperature difference of the ground at 15 cm depth between SALIX and TUNDRA, corrected for air temperature. Negative values indicate that at equal air temperature, the ground temperature is colder at SALIX than at TUNDRA. The dashed horizontal lines are the 0 °C lines, added as visual aids.

**Extended Data Fig. 2:**
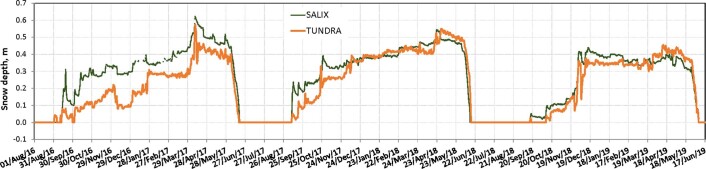
Snow depth at SALIX and TUNDRA for the 3 years studied. Observed snow depth at SALIX and TUNDRA for the 3 years studied.

**Extended Data Fig. 3:**
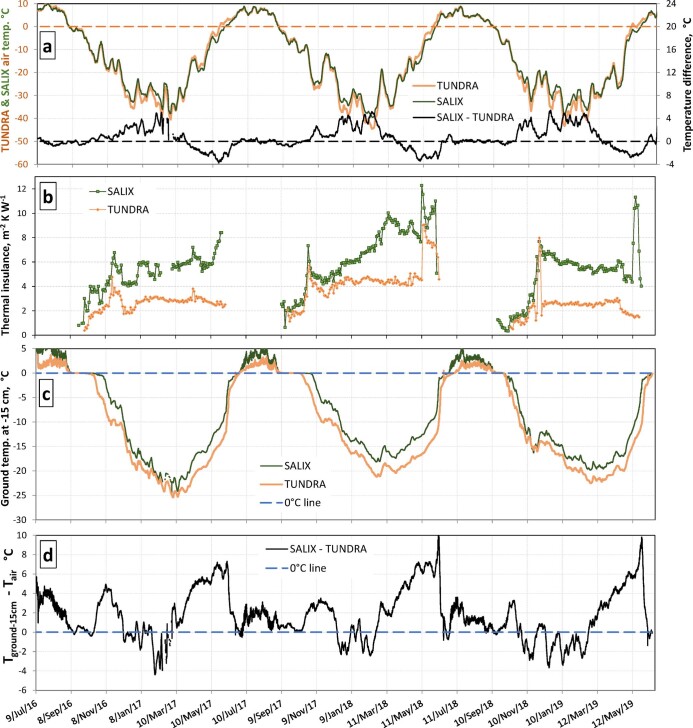
Thermal variables at SALIX and TUNDRA for the 3 years studied. (a) One-week running mean air temperature and one-week running mean temperature difference between both sites. (b) Snowpack thermal insulance. (c) One-week running mean temperature of the ground at 15 cm depth at both sites. (d) Temperature difference of the ground at 15 cm depth between SALIX and TUNDRA, corrected for air temperature. Horizontal dashed lines are visual aids for 0 °C temperatures.

Three years (2016–2019) of meteorological, snow and ground data were obtained, including snow and ground thermal conductivity and temperature, and ground liquid water content, all of these variables at several levels. Field measurements of snow and ground properties were made in spring and summer 2016 to 2019, including measurements of ground thermal conductivity and density. Ground granulometric analyses were performed in the laboratory (Supplementary Fig. 2). Here we focus on the 2018–2019 snow season. Other years revealed similar processes. Figure 2 shows the evolution of snow depth in 2018–2019. Snow onset date is earlier at SALIX because it is closer to the mountains and the orographic effect is more marked, but no major difference appears between the two sites. Data for other years are in Extended Data Fig. 2. The air temperature is colder at TUNDRA in winter (Fig. 3a) because of katabatic flow in the river bed. SALIX is colder in spring probably because of the 3° slope with northerly aspect, which reduces radiative heating. Data similar to all panels of Fig. 3 are shown for the 3 years of study in Extended Data Fig. 3. The hourly wind speed averaged over the study was 1.98 m s^−1^ at TUNDRA and 1.43 m s^−1^ at SALIX. Values seldom exceeded 10 m s^−1^.

**Extended Data Fig. 4:**
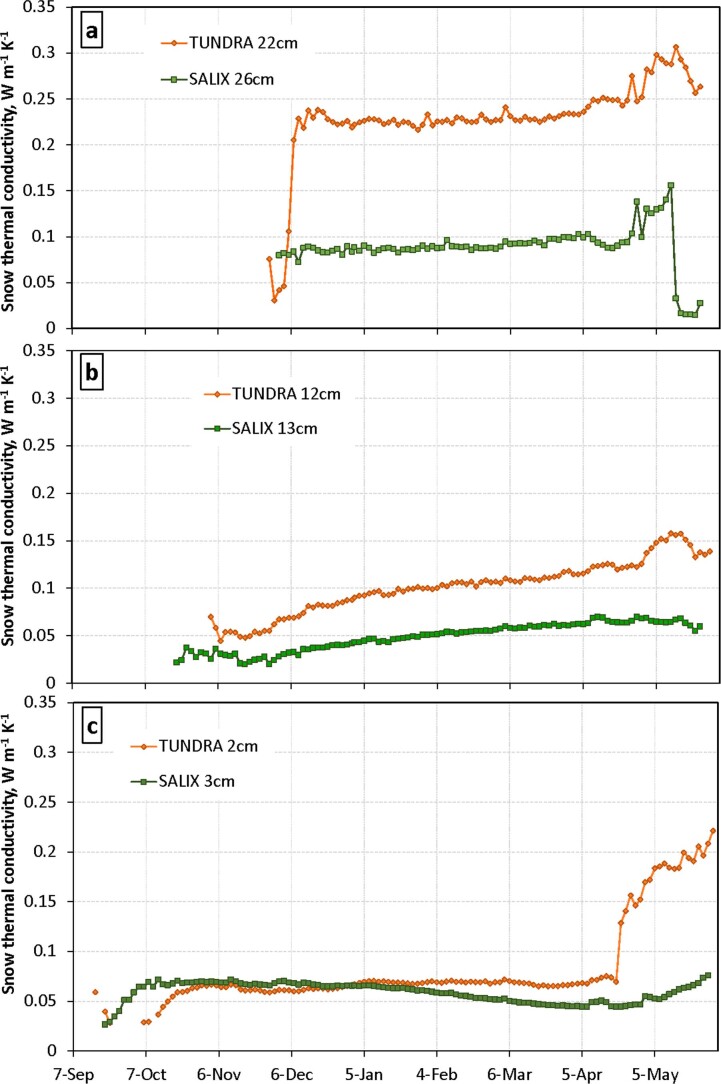
Snow thermal conductivities at SALIX and TUNDRA for 2108-2019. (a) Upper level, with needle probes at 22 cm (TUNDRA) and 26 cm (SALIX) heights. The conductivity is almost always much higher at TUNDRA. (b) middle level at 12/13 cm. Likewise, the conductivity is always much higher at TUNDRA. (c) lower level 2/3 cm. The conductivity is similar at both sites until mid-January, when it starts decreasing at SALIX.

Figure 3b reports the evolution of the snowpack thermal insulance *R*_T_, calculated (see Methods) using the thermal conductivity profiles shown in Extended Data Fig. 4. The thermal effects of shrubs are not included when we consider snowpack insulance. Given the much greater thermal insulance at SALIX, the temperature of the ground is expected to be much warmer there^7^. Between 1 November 2018 and 1 April 2019, the ground temperature at 15 cm depth is on average 1.97 °C warmer at SALIX (Fig. 3c). However, over that period the air temperature at SALIX is 2.56 °C warmer (Fig. 3a), meaning that for an equal air temperature, the ground temperature is 0.59 °C colder at SALIX (Fig. 3d), despite the much more insulating snowpack. The presence of shrubs therefore cools the ground. Figure 3d shows that between 24 October 2018 and 24 February 2019, the air–ground temperature difference is lower at SALIX, implying more efficient ground cooling there. On 18 December 2018, the ground cooling is 3.66 °C greater at SALIX than at TUNDRA. Between 13 October and 21 November, the ground cools by 15.2 °C at SALIX, much more that the 11.9 °C cooling at TUNDRA (Fig. 3c), again despite the greater snow thermal insulance at SALIX. Similar conclusions are reached for the snow–ground interface (Supplementary Fig. 3). The first line of Table 1 sums up ground temperature differences between SALIX and TUNDRA for various time periods.

**Table 1:** Time-averaged differences in ground temperature

	24 Oct–24 Feb	1 Dec–20 Apr	24 Feb–10 May	20 Apr–10 May	24 Oct–10 May
Observations: effect of snow + shrubs^a^					
SALIX − TUNDRA (°C)	−1.21^b^	+0.27	+3.39	+5.34	+0.53
Simulations at SALIX: effect of thermal bridging only^c^					
(Snow + shrubs) − (snow only) (°C)	−1.12	−1.40	−0.67	+1.02	−0.95

Values are for 15 cm depth, for specified time periods. The first line of values compares observations between SALIX and TUNDRA. The second line of values compares finite element simulations at SALIX for the cases with and without shrubs. Both lines are not expected to show similar values as snow in the second line has properties for the SALIX case, whereas in the first line the data include changes due to the differences in snow properties between TUNDRA and SALIX.

^a^As air temperatures are not identical at both sites, ground temperature minus air temperature values have been used.

^b^Negative values in observations indicate colder ground at SALIX.

^c^Negative values for simulations indicate a cooling effect of shrubs.

**Extended Data Fig. 5:**
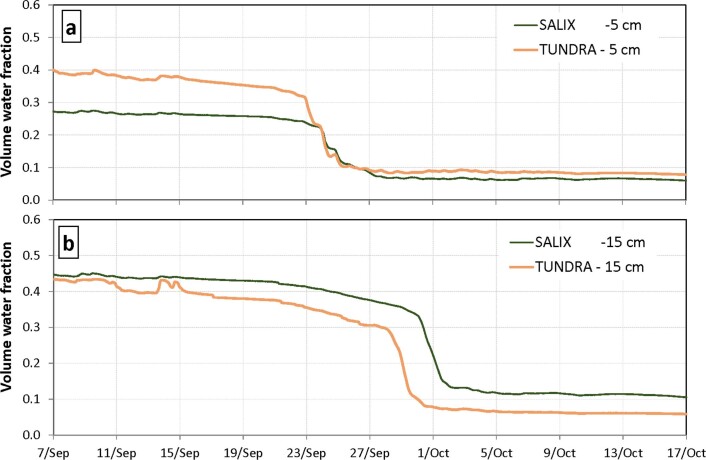
Soil volume liquid water content. Data at 5 and 15 cm depth at SALIX and TUNDRA in fall 2018 are presented.

Ground properties affect the permafrost thermal regime^10^ and were investigated. Extended Data Fig. 5 shows that in autumn 2018 the volume liquid water content at 5 cm depth was higher at TUNDRA (38% versus 27% at SALIX), whereas at 15 cm depth it was slightly higher at SALIX (44% versus 41% at TUNDRA). At 5 cm depth, freezing was on 25 September at both sites. At 15 cm depth, freezing was on 1 October at SALIX, 2 days later than at TUNDRA. Slight differences in ground properties may have contributed to this delay, which in any case is too small to substantially affect our subsequent discussion of temperature changes after freezing. Ground water data for 3 years are in Supplementary Fig. 4. Ground thermal conductivities (Supplementary Fig. 5) showed essentially one value when thawed and one value when frozen. Ground density profiles (detailed in the Supplementary Material) from several pits at both sites revealed similar values in the range 1,000–1,850 kg m^−3^, increasing with depth down to the freezing level. Thermal conductivity values increased from 0.25 to 1.7 W m^−1^ K^−1^ at 25 cm depth. Ground granulometry (Supplementary Fig. 2) identified sandy silt and silty sand with fairly similar size distributions at both sites. In summary, given variations between nearby spots at each site, there is no notable difference in ground density, thermal conductivity and granulometry between TUNDRA and SALIX.

**Extended Data Fig. 6:**
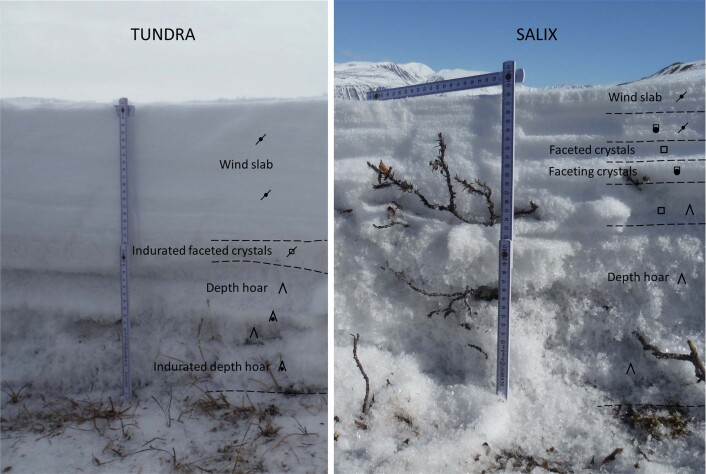
Photographs of snowpits at TUNDRA and SALIX. These pictures were taken in May 2015, showing the difference in stratigraphy and in particular the greater depth hoar thickness at SALIX.

Field observations of snow were made near both sites in mid-May 2019. Density profiles from two pits at each site are shown in Supplementary Fig. 6. The average density of the SALIX pits was 267 kg m^−3^ versus 317 kg m^−3^ at TUNDRA, 19% greater than SALIX. This is similar to earlier observations at similar spots in 2017 (17% greater at TUNDRA^19^) and in 2015 (19% greater at TUNDRA^7^). Because density and thermal conductivity are positively correlated^20–23^, the greater density observed at TUNDRA is consistent with monitored thermal conductivities. All pits had a basal depth hoar layer 10–25 cm thick, slightly denser at TUNDRA than at SALIX. Upper layers at SALIX were mostly faceted crystals with an occasional thin top wind-packed layer. At TUNDRA, the upper snowpack was mostly wind slabs. Photographs of the May 2019 snowpits are not available. Instead, Extended Data Fig. 6 shows photographs of May 2015 snowpits, when snow conditions were similar^24^.

**Extended Data Fig. 7:**
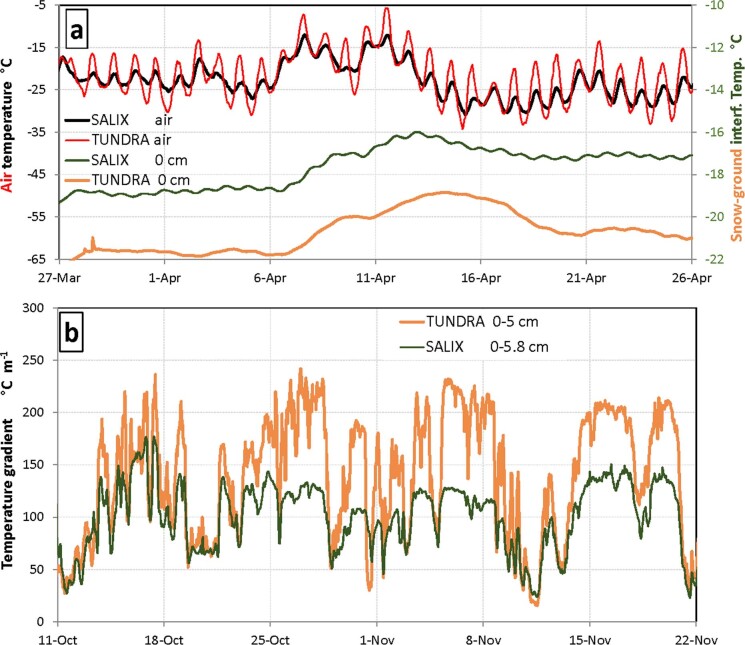
Comparison of thermal data at TUNDRA and SALIX. (a) Snow-ground interface temperatures and air temperatures at SALIX and TUNDRA for March-April 2019 during a sunlit period. Note the greater air temperature amplitude at TUNDRA, which contrast with the greater snow-ground temperature amplitude at SALIX; (b) temperature gradient in the basal snow layer (between 0 and 5 cm height at TUNDRA and 0 and 5.8 cm height at SALIX) at TUNDRA and SALIX between 11 October and 22 November 2018.

Additional data point towards a heat transfer process other than conduction through snow. Diurnal temperature variations in spring (Extended Data Fig. 7a) are propagated through the snowpack to the snow–ground interface much faster and with greater amplitude at SALIX than TUNDRA, despite the much more insulating SALIX snowpack. Extended Data Fig. 7b shows that the temperature gradient ∇*T* in the basal layer of the TUNDRA snowpack is almost always much greater at TUNDRA than at SALIX between 11 October and 22 November. The thermal conductivity, *k*_eff_, of the basal snow layer (Extended Data Fig. 4c) is similar at SALIX and TUNDRA, so that the heat flux *F* = −∇*T* × *k*_eff_ through the basal snow layer is much greater at TUNDRA than at SALIX. Despite this greater heat flux, the ground cools more slowly at TUNDRA than at SALIX during that period (Fig. 3c). These considerations lead to the conclusion that conductive heat fluxes through snow only cannot explain observations and that another process comes into play at SALIX.

We hypothesized that heat conduction through frozen shrub branches could explain our observations and tested this using simulations. We (1) simulated heat transfer through snow only to verify that it could explain the temperature data at TUNDRA but not at SALIX; and (2) performed finite element simulations of heat transfer through both snow and shrubs at SALIX.

## Thermal bridging through shrubs

**Fig. 4:**
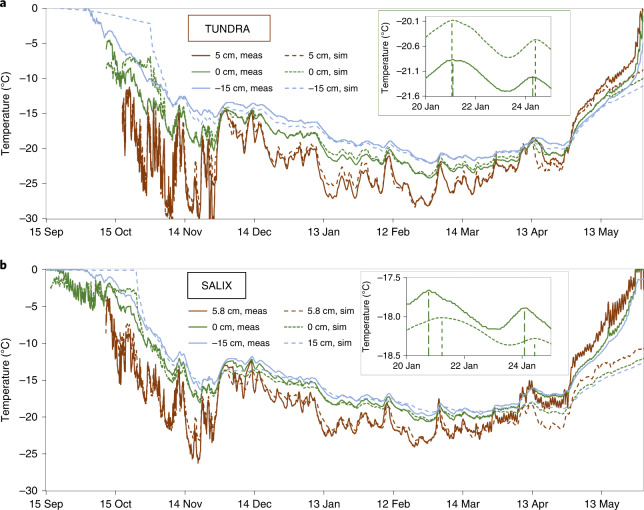
Comparison of measured and simulated snow and ground temperatures. Simulations were performed with the MFM at 0 and 5 or 5.8 cm heights and at 15 cm depth. **a**, At TUNDRA. **b**, At SALIX. In both cases, an inset illustrates the quality of simulation of the phase at 0 cm. The shift is less than 2 h at TUNDRA and more than 10 h at SALIX. meas, measured; sim, simulated. Source data

The thermal regime of the snow and ground at TUNDRA was simulated with the Minimal Firn Model^25,26^ (MFM) using measured values of thermal variables (Supplementary Table 1). Snow thermal conductivity values were multiplied by 1.2 to account for the underestimation of the needle probe method^27^. The model simulates the snow and ground temperatures mostly within less than 1 °C (Fig. 4a), except during ground freezing in September, as the model does not simulate the water phase change. An artificially high ground heat capacity was used until freezing completion (Supplementary Table 1). Once the resulting perturbation is over, simulations reproduce data well. Furthermore, the phase of the temperature changes is reproduced within a few hours (Fig. 4a, inset). The simulations are excellent in spring until mid-May, when snowmelt dramatically modifies all snow properties, which is not simulated.

At SALIX, to satisfactorily reproduce temperature data (Fig. 4b), measured *k*_eff_ values had to be multiplied by 1.4 for the period until 31 December and then by 1.7. Furthermore, thermal waves are propagated about 12 h later in simulations than in measurements at the snow–ground interface. This confirms that a heat transfer process other than conduction in snow is taking place.

Faster cooling can be simulated by lowering the ground density and/or heat capacity or increasing the ground thermal conductivity^10^ by over 60%, which is inconsistent with field measurements. Furthermore, thermal waves were still propagated 12 h late. Ground properties very different from measured values therefore cannot explain observations. Heat transfer through shrub branches must be invoked.

**Extended Data Fig. 8:**
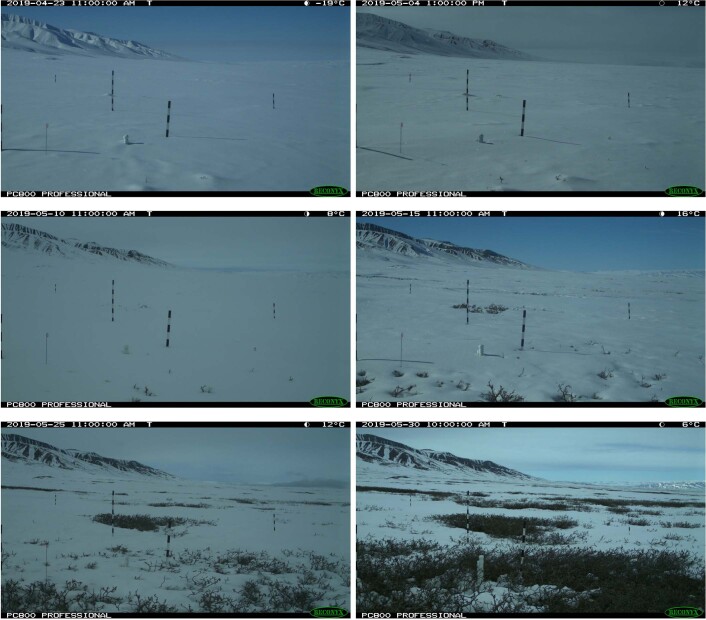
Time lapse images of the SALIX site. This shows the progression of branch protrusion above the snow. On 23 April, near peak snow height, essentially no branches protrude above the snow. On 4 May, still very few branches are visible. On 10 May, a few branches become visible. On 15 May, an important number of branches are visible and signs of melting around them are starting to appear. On 25 May, melting is substantial around the branches and snow height at the front snow stake has decreased 8 cm relative to 23 April. On 30 May, most of the snow is melted. Meltout at the plastic post was on 3 June. About 4 cm of snow fell on 4 June and final meltout for the visible flat area was on 9 June.

Starting around mid-April, simulated temperatures are much lower than measured ones at SALIX: 3.5 °C lower at 15 cm depth on 10 May, before the onset of melting on 12 May. Another heat transfer process therefore now warms up the snow and the ground. Because branches do not protrude above the snow until 12 May (see time-lapse images, Extended Data Fig. 8), decreased albedo, evidenced for tall shrubs and which accelerates snowmelt^28,29^, does not operate efficiently here. We instead propose that solar radiation transmitted through the snow heats up buried branches, and this heat diffuses to the ground through the branches. Starting on 18 April, daily radiation maxima exceed 500 W m^−2^ and 24-h averages reach 200 W m^−2^ (ref.^30^). The *e*-folding depth for visible radiation in Arctic snow is 5–15 cm (ref. ^31^). More than 50% of incident radiation energy is in the visible, meaning that a daily average radiative flux of about 50 W m^−2^ is likely in snow at SALIX at 10 cm depth. Arctic shrub branches have an albedo <0.1 in the visible^32,33^, so shrubs probably absorb a substantial amount of energy.

**Fig. 5:**
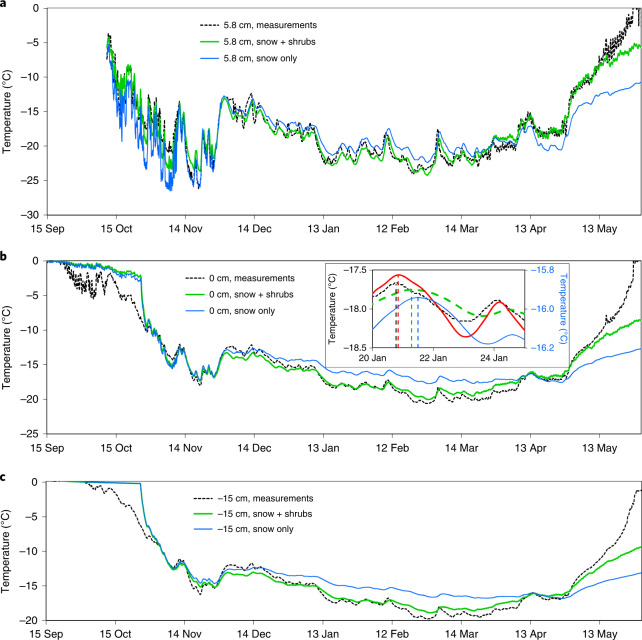
Finite element simulations of temperatures at SALIX. Simulated snow and ground temperatures resulting from heat transfer through a snow cover with and without shrubs are compared. Measured temperatures are also shown. **a**–**c**, At 5.8 cm height in the snow (**a**), at the snow–ground surface (**b**) and in the ground at 15 cm depth (**c**). In **b**, the inset shows the quality of the phase simulation, with the dotted vertical lines marking the maxima of temperature. The green dashed curve is the temperature of a point halfway between two shrubs, while the red curve is the temperature in the middle of a shrub (Extended Data Fig. 9), to which 2 °C was added to facilitate comparison. The right-hand temperature scale (in blue) is for the blue curve only. Source data

We therefore performed finite element simulations of heat transfer through the (snow + shrubs) system, considering heat conduction through branches and radiation absorption by buried branches in spring. The results of Fig. 5 are for a spot halfway between two shrubs (green dot, Supplementary Fig. 12). Comparing simulations with and without shrubs shows that the maximum shrub cooling effect of the ground at 15 cm depth is 2.29 °C on 27 February (Extended Data Fig. 10). For the snow–ground interface, the maximum cooling is 2.30 °C, also on 27 February. In spring, however, shrubs warm the ground, by 1.91 °C on 10 May (Extended Data Fig. 10). Beyond that date, simulations and measurements start diverging because snow temperature nears 0 °C and our simulations do not consider melting.

**Extended Data Fig. 9:**
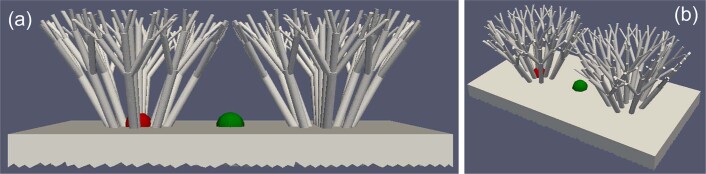
Schematic of the shrub geometry used in the finite element simulations. (a): side view. (b): top view. Shrubs are 38 cm high and the shrubs centers are 64 cm apart. The green dot between shrubs corresponds to the spot where the snow-ground interface temperature has been calculated for the green curves in Fig. 5 in the main text, including the inset of Fig. 5b in the main text. The red dot in the shrub is the spot relevant to the red curve in the inset of Fig. 5b in the main text.

Regarding the phase of the temperature changes (inset, Fig. 5b), simulations that include just snow lag measurements by 16 h. Simulations with both snow and shrubs still lag by 12 h. However, simulations for a spot in the middle of a shrub rather than halfway between two shrubs (respectively red and green dots in Extended Data Fig. 9) reduced the lag to <2 h. Because our interface temperature sensor is about 10 cm from the middle of a shrub, we conclude that our measurements are well simulated by including conduction through frozen shrub branches.

**Extended Data Fig. 10:**
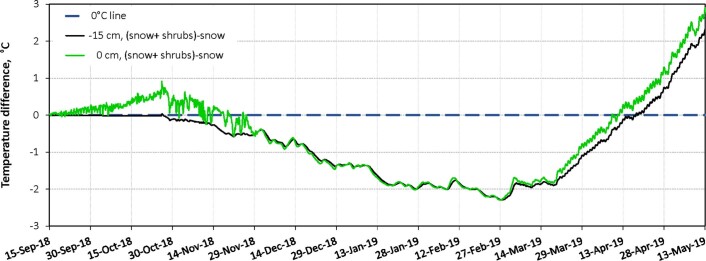
Finite element simulations of the effect of thermal bridging through shrub branches on temperature. Simulations are shown for the ground at 15 cm depth and for the snow-ground interface (0 cm depth). This plot shows the temperature simulated with both snow and shrubs minus the temperature simulated with snow only. From December through April, these differences are negative, indicating that thermal bridging through shrub branches cools the interface and the ground. During about the last 30 days of the plot, radiation absorption by shrubs branches and the resulting branch heating warm up the interface and the ground.

The simulated cooling of the ground at 15 cm depth by thermal bridging through shrub branches lasts until 20 April (Fig. 5c and Extended Data Fig. 10). Between 1 December and 20 April, the average cooling effect of thermal bridging is 1.40 °C. Between 20 April and 10 May, the average warming is 1.02 °C. These values are summed up in the second line of Table 1. Overall, between 24 October (when the effect of thermal bridging starts to manifest itself; Extended Data Fig. 10) and 10 May, the average effect of thermal bridging is a cooling of 0.95 °C. However, as our model does not consider snowmelt, we cannot perform simulations beyond that date. Simulating warming due to protruding branches and their shading effect would also be required. In any case, we demonstrate that thermal bridging through shrub branches substantially modifies the yearly evolution of the ground temperature, with important cooling in winter and important warming in spring. Despite the spring warming caused by thermal bridging, meltout at SALIX and TUNDRA were nearly simultaneous: 7 June at TUNDRA and 9 June at SALIX, suggesting that cooling effects such as branch shading may also take place in spring.

## Global impact and GHG emissions

Thermal bridging through shrubs, detected here, leads to a 2.3 °C maximum ground cooling (Extended Data Fig. 10). In most cases, shrubs increase snow height^6,8,9,34^ and decrease snow thermal conductivity^6,7^, leading to ground warming. Radiation absorption by buried branches also lead to warming. The snow season averaged effect of shrubs, resulting from thermal bridging, radiation absorption and modified snow properties, may often be warming, as reported earlier^6,9^. Cooling by thermal bridging, although previously undetected, may however be an Arctic-wide process. A recent study^9^ reports warmer ground temperature under tall shrubs than under lower vegetation in March at a 68.7 °N site, consistent with an overall warming effect of shrubs. However, in October, the authors report colder temperatures and faster ground cooling under tall shrubs. This may tentatively be interpreted as an effect of thermal bridging through shrub branches. The manifestation of this effect may be highly variable as it is affected by shrub size. Taller shrubs may indeed lead to greater snow height, but their thicker stems may cool the ground efficiently in autumn before substantial snow accumulation. A complicating effect is that thermal bridging effects may be concealed by ground properties, because litter accumulation under shrubs^35,36^ lowers ground thermal conductivity, which slows down cooling. Other effects such as shrub-induced snowmelt in autumn^37^ may also intervene and cool the ground in autumn by reducing the insulating snowpack. Detecting and quantifying thermal bridging in a given setting therefore requires extensive measurements, including snow and ground thermal properties. Temperature and snow height are not sufficient for detecting with certainty, quantifying and reliably simulating thermal exchanges.

The accelerated early winter cooling by thermal bridging blocks nutrients recycling and favours litter accumulation^38^, while the opposite effect is expected in spring. The balance between winter and spring effects is expected to be highly variable depending on site and shrub properties. Sites with northern aspect will be less affected by spring radiative heating, especially given the low sun angles in the Arctic, and thermal bridging is expected to lead to enhanced litter accumulation there.

Changes in the ground thermal regime affect its GHG emissions. Quantifying this effect is essential for climate projections^3^, but at present accurate data on shrub geometry and current and future distributions are insufficient. However, because GHG emissions by frozen grounds greatly increase with increasing temperatures^15–17^, the spring warming due to absorption of solar radiation by buried shrub branches will probably increase GHG emissions more than the decrease caused by cooling due to thermal bridging in winter. While the magnitude of these processes remains uncertain, they represent yet other processes not accounted for in climate projections^3^, which may further increase the fluxes of GHG to the atmosphere from permafrost in a warming climate that favours shrub expansion.

## Methods

At TUNDRA, we monitored standard meteorological data (air temperature and relative humidity at 2.3 m height, wind speed, long-wave and short-wave radiation fluxes with a CNR4/CNF4 instrument from Kipp & Zonen), snow depth with an ultrasonic gauge, snow surface temperature using an infrared sensor, snow temperature with thermistors and snow thermal conductivity with TP08 heated needle probes from Hukseflux. Soil temperature and volume water content were monitored with 5TM sensors from Decagon. Soil thermal conductivity was measured with TP08 heated needle probes^30^. At SALIX, similar instruments were deployed, except snow surface temperature, which was not monitored. Regarding radiation, only downwelling short-wave was measured there.

For air temperature, Rotronic HC2A-S3 sensors were used, which have an accuracy of 0.1 °C. Having no shift between both sensors at SALIX and TUNDRA is important. In summer, when the valley air temperature is homogenized by convective mixing, the temperature difference between both sites is very low. Between 15 July and 15 September 2018, the weekly averaged temperature difference (Fig. 3a) remained in the range −0.29 to +0.30 °C. The average difference over that period was 0.060 °C, giving us confidence in the reliability of the air temperature measurements. The 5TM sensor has a manufacturer-stated accuracy of 1 °C and a resolution of 0.1 °C. Our sensors showed a time-stable offset of 0.1–0.5 °C, which was easily corrected using the temperature value during zero-curtain periods. Ground temperatures are thus accurate within <0.1 °C. Snow temperatures were measured with Pt100 thermistors, and any offset was corrected using data during snowmelt, when snow temperature remains at 0 °C for at least a few hours each day. Snow surface temperature at TUNDRA was measured with an IR120 infrared sensor from Campbell Scientific, which sensed radiation in the 8–14 µm wavelength range. The manufacturer’s stated accuracy is 0.2 °C. During snowmelt, the maximum recorded temperature was 0.3 °C, so a slight offset is possible. However, as we only have a surface temperature measurement at TUNDRA and use its value at SALIX after correction, any use of the IR120 data will not affect our temperature comparison between both sites. Snow depth was measured with a SR50A gauge from Campbell Scientific, with a stated accuracy of 1 cm.

In snow, the TP08 needles were placed on a vertical post at 2, 12 and 22 cm heights at TUNDRA, and at 3, 13 and 26 cm heights at SALIX. The heights were a bit greater at SALIX because in May 2015, a year prior to the installation of SALIX in July 2016, we observed that the snowpack at SALIX was a bit thicker^7^ and we wanted to probe equivalent layers. At SALIX, we ensured that the TP08 needles were not in direct contact with shrubs’ branches. At TUNDRA, another vertical post about 2 m from the TP08 post held thermistors at 0, 5, 15, 25 and 35 cm heights. At SALIX, thermistors were placed on the TP08 post at 0 and 5.8 cm heights. In the ground, TP08 needles were placed at 10 cm depth at TUNDRA, and 5 and 15 cm depth at SALIX. Ground temperature and volume water content sensors were placed at 5, 15 and 30 cm depth at SALIX, and at 2, 5, 10, 15 and 21 cm depth at TUNDRA. The shallower thawed layer at TUNDRA (20–25 cm in mid-July, versus 35–45 cm at SALIX) prevented the installation of deeper sensors. Note that the thicker thawed layer at SALIX is not necessarily an indication of greater heat conduction in summer, as SALIX is at the base of an alluvial fan with substantial water flow that may contribute to summer ground thaw by heat advection. Time-lapse cameras taking several pictures a day were placed at both sites with the TP08 posts in their field of view. Photographs of both sites showing the posts are included in Supplementary Fig. 1. Field observations of snow stratigraphy and measurements of vertical density profiles were performed near the TUNDRA and SALIX sites in mid-May in 2017, 2018 and 2019. Density profiles were measured with a 3-cm vertical resolution by weighing snow samples taken with a 100-cm^3^ density cutter. Field observations of soil density, thermal conductivity and liquid water content were performed at various locations around both study sites in early July 2016 and 2017. Soil samples were brought back to Laval University for granulometric analysis using a laser particle size counter. Soil properties at TUNDRA are detailed in ref. ^30^.

Thermal conductivity, *k*_eff_, was measured by heating the TP08 needle for 100 s and by monitoring the temperature rise, as detailed in ref. ^39^. Briefly, the plot of the temperature rise as a function of log(time), after a transition period of about 15 s, yields a straight line whose slope is inversely proportional to *k*_eff_. One measurement is performed every two days to minimize the energy input to the snow and because *k*_eff_ variations are usually slow. Because the measurement requires heating, it is disabled if the snow temperature is above −2.5 °C to avoid melting and irreversible modification of the snow structure. Measurements are therefore often not available in late spring. Measurements were discarded when the quality of the plot was insufficient, as detailed in ref. ^39^. This was infrequent for snow but was frequent for the ground, especially when frozen, because the same heating power for snow and ground had to be used with our setup, and this power was optimized for snow. Frozen ground often has a thermal conductivity around 2 W m^−1^ K^−1^, so the heating of the needle is low and the quality of the plot not as good as for less conductive media such as snow.

The thermal insulation properties of the snowpack are best summed up by its thermal insulance *R*_T_ (ref. ^26^), with units of m^2^ K W^−1^. *R*_T_ simply relates the heat flux through the snowpack *F* to the temperature difference between its surface and its base, *T*_top_ − *T*_base_:

1 $$F = - \frac{{T_{{\mathrm{top}}} - T_{{\mathrm{base}}}}}{{R_{\mathrm{T}}}}$$

For a plane-parallel layered medium, *R*_T_ is defined as:

2 $$R_{\mathrm{T}} = \mathop {\sum}\limits_i {\frac{{h_i}}{{k_i}}} ,$$

where *h*_*i*_ and *k*_*i*_ are the height and thermal conductivity of layer *i*, respectively.

*R*_T_ time series were calculated using the thermal conductivity data. The snowpack was divided into three layers according to stratigraphies observed in May, with layer boundaries close to 10 and 20 cm heights. The thermal conductivity of each layer was assumed to be homogeneous.

Simulations of the snow and ground temperature evolutions were performed with the MFM detailed in ref. ^25^, which uses a Crank–Nicolson scheme to calculate heat propagation through the snow and soil. The model driving data were hourly measurements of snow surface temperature at TUNDRA. At SALIX, snow surface temperature was not available. We used TUNDRA surface temperature corrected by the difference in air temperature between both sites. The snowpack was divided into three homogeneous layers corresponding to the 0–10, 10–20 and 20–38 cm heights. The ground was divided into two layers between 0–10 and 10–500 cm depths. Ground densities *ρ*_g_ were measured at several spots close to our study sites using a cylindrical density cutter. In MFM simulations, *ρ*_g_ values were adjusted within the measured ranges to optimize the fits (Supplementary Table 1). Specific heat *C*_p_ values were likewise adjusted around values following the data of ref. ^40^. Ground thermal conductivities *k*_g_ were based on measurements. It is important to note that the rate of cooling of the ground depends on its thermal diffusivity *α*_g_ *= k*_g_*/*(*ρ*_g_*C*_p_), so different combinations producing the same *α*_g_ values yield similar fits. Measured snow depths were used, simplified by using a step-wise function with seven time intervals over the season at TUNDRA and eight intervals at SALIX. It has been proposed that snow thermal conductivity *k*_s_ measurements using heated needle probes may show a systematic negative bias of 10–50%, depending on snow type^27^. We therefore used our measured snow thermal conductivity values multiplied by 1.2. MFM does not simulate water phase changes and thus cannot simulate the ground zero-curtain period, that is, the period during which the ground remains at 0 °C while its water freezes. Instead, we tested that using a ground specific heat value of 600 kJ kg^−1^ K^−1^ during the zero-curtain period reproduced measurements well.

Modelling of heat transfer through shrub branches was done by finite element simulations using the open source ElmerFEM software^41^. The shrub geometry used is illustrated in Extended Data Fig. 9. It attempted to reasonably mimic observations (Supplementary Fig. 1). Three levels of branches were used, with diameters of 3, 2.3 and 1.36 cm. The branches extend to a height of 38 cm and the centre of both shrubs are 64 cm apart.

The finite element mesh was generated using the Gmsh software^42^. The simulation was run with an hourly time step and forced with snow surface temperature, similarly to the MFM simulations. The absorption of solar radiation by the shrub branches was simulated by adding a heat source in the upper branches. The heat source was chosen so that the total energy absorbed by the shrubs equals 0.25% of the total downwelling short-wave flux over the snow surface. This 0.25% fraction was adjusted in order to reproduce the snow and ground warming in spring.

The fomulations required to specify a value for the thermal conductivity of shrub branches. There does not appear to be any measurement of the thermal conductivity of live or fresh wood at subfreezing temperatures. A study^43^ measured the thermal conductivity and diffusivity of samples from temperate and tropical wood species, wet or dry. The authors found that the axial thermal properties were much larger than radial and tangential ones. For ash (*Fraxinus*), the one temperate species studied, they measured *k*_wood_ = 0.9 W m^−1^ K^−1^ and *α*_wood_ = 7.1 × 10^−7^ m^2^ s^−1^. Other studies all found that axial conductivity was greatest and measured axial conductivity values between 0.5 and 1 W m^−1^ K^−1^ for wet wood^44–46^. Because ice thermal conductivity is four times that of water, branch freezing is likely to lead to an increase in thermal conductivity. Supercooling does take place in shrub branches but not does not seem to reach −20 °C (refs. ^47,48^), to which the shrubs of interest here are exposed. Cold-exposed shrubs have developed adaptations where freezing of water takes place in the extracellular space^47,48^, but ice does form so that an increase in shrub thermal conductivity upon freezing is likely and we thus tested the impact of shrubs whose branches have thermal conductivity values of 1 and 2 W m^−1^ K^−1^. For the shrub geometry used, the best results were obtained for a value of 2 W m^−1^ K^−1^. In comparison, the data obtained here on snow thermal conductivity show all winter values to be <0.1 W m^−1^ K^−1^ (Extended Data Fig. 4). Even if a multiplicative factor of 1.2 is applied to account for the underestimation by the needle probe method, it appears that shrub branches have a thermal conductivity about 20 times as large as that of snow. It is possible that frozen wood thermal conductivity is even higher than 2 W m^−1^ K^−1^, in which case thinner branches could be used to simulate the same thermal effects. We used a wood specific heat value of 1,200 J kg^−1^ K^−1^ (ref. ^49^), but tested that a value of 2,000 did not produce any detectable change in the simulation. The wood density used was 900 kg m^−3^, but changing this value had no impact on simulations either.

## Online content

Any methods, additional references, Nature Research reporting summaries, source data, extended data, supplementary information, acknowledgements, peer review information; details of author contributions and competing interests; and statements of data and code availability are available at 10.1038/s41561-022-00979-2.

## Supplementary information

Supplementary Information Supplementary Figs. 1–6, description of field site and soil properties, and Supplementary Table 1.

## Data Availability

All meteorological, snow and soil data for the TUNDRA site are detailed in ref. ^30^ and the data files are available at 10.5885/45693CE-02685A5200DD4C38 (ref. ^50^). The data for the 2018–2019 period, subject of this paper, are available on the Nordicana repository at 10.5885/45786CE-3A2A2BFB295D4BE2 (ref. ^51^). Source data are provided with this paper.

## Source data

Source Data Fig. 4 Temperature time series.
Source Data Fig. 5 Temperature time series.

## Code availability

The MFM code is available at http://github.com/ghislainp/mfm. The specific driver to perform the simulations presented here is available at 10.5281/zenodo.6504950.
