# Supplementary material for: Permafrost cooled in winter by thermal bridging through snow-covered shrub branches
Source: Nat Geosci. 2022 Jul 7;15(7):554–60. doi: 10.1038/s41561-022-00979-2 (PMC9279148; doi:10.1038/s41561-022-00979-2)
Supplement: Supplementary file 1 — Supplementary Figs. 1–6, description of field site and soil properties, and Supplementary Table 1. [file 41561_2022_979_MOESM1_ESM.pdf]

nature portfolio

https://doi.org/10.1038/s41561-022-00979-2

Supplementary information

Permafrost cooled in winter by thermal bridging through snow-covered shrub branches

In the format provided by the authors and unedited

Supplementary Material for

PERMAFROST COOLED IN WINTER BY THERMAL BRIDGING THROUGH BRANCHES OF SNOW-COVERED SHRUBS

Florent Domine*, Kevin Fourteau, Ghislain Picard, Georg Lackner, Denis Sarrazin, Mathilde Poirier

*Corresponding author. Email: florent.domine@gmail.com

Details on study site

TUNDRA is at the bottom of the valley while SALIX is up on a bank about 5 m higher than the bed of the braided glacial river. At TUNDRA, the vegetation consists of moss and grasses dominated by *Dupontia fisheri*, *Carex aquatilis* and *Eriophorum scheuchzeri*[1]. At SALIX, vegetation at the very spot where snow and ground instruments were deployed (Fig. 1) consists mostly of *Salix richardsonii* with a very thin moss understory. Basal branches often have a diameter of about 2 cm. Some occasional *Salix arctica* and *S. reticulata* were also observed. In other nearby spots with less dense shrubs (Fig. 1), species such as *Arctagrostis latifolia* and *Oxytropis arctica* were seen, as well as some bare ground. Photographs of shrubs showing their architecture are shown in Fig. 1. The granulometry of ground samples at both sites and at several depths is shown in Fig. 2.

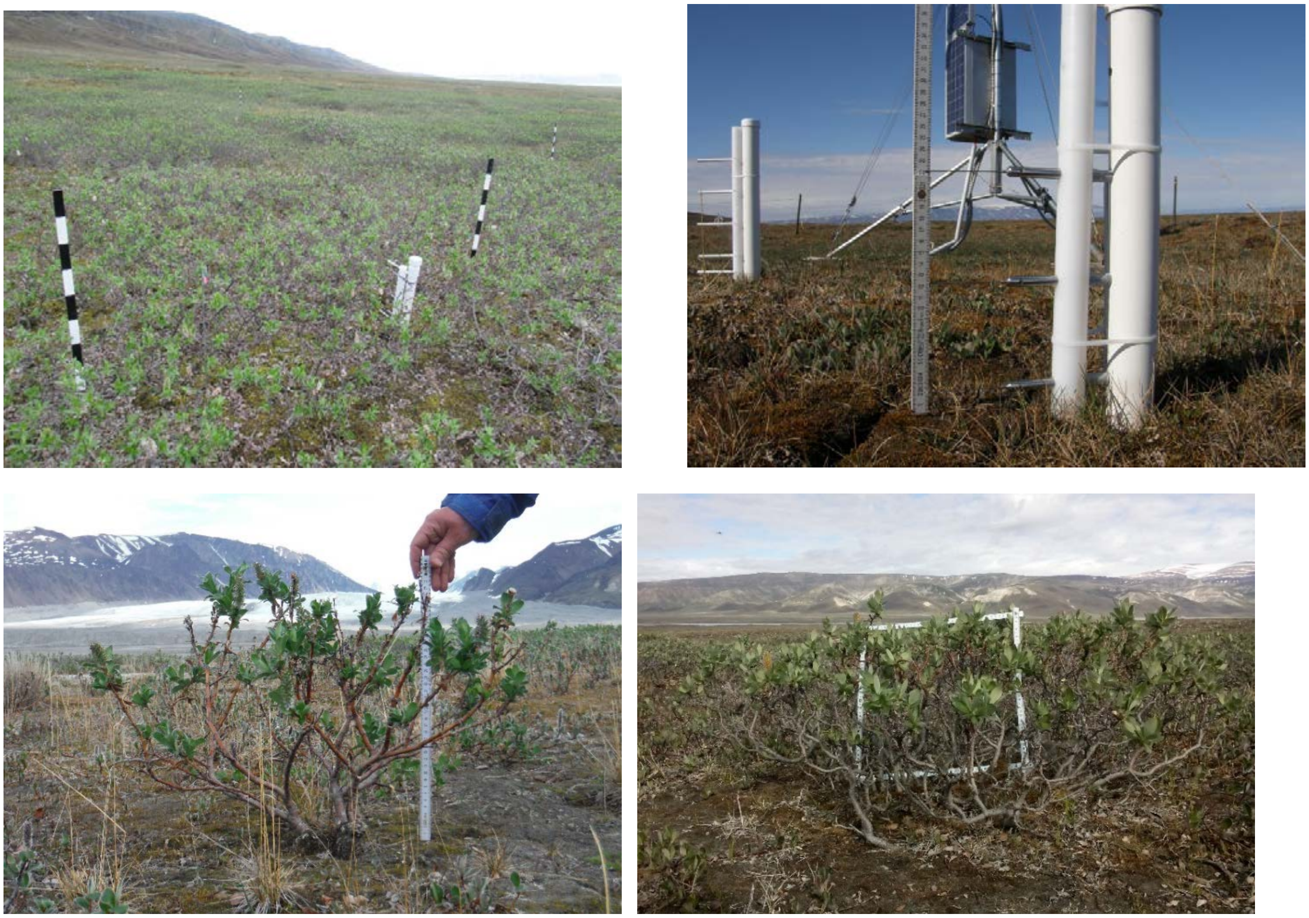

Fig. 1. Photographs of the sites with snow sensors. Top: photographs of the SALIX (left) and TUNDRA (right) sites showing the post supporting the TP08 heated needle probes which measure snow thermal conductivity. Bottom: photographs of shrub structure. Shrub height is approximately 40 cm.

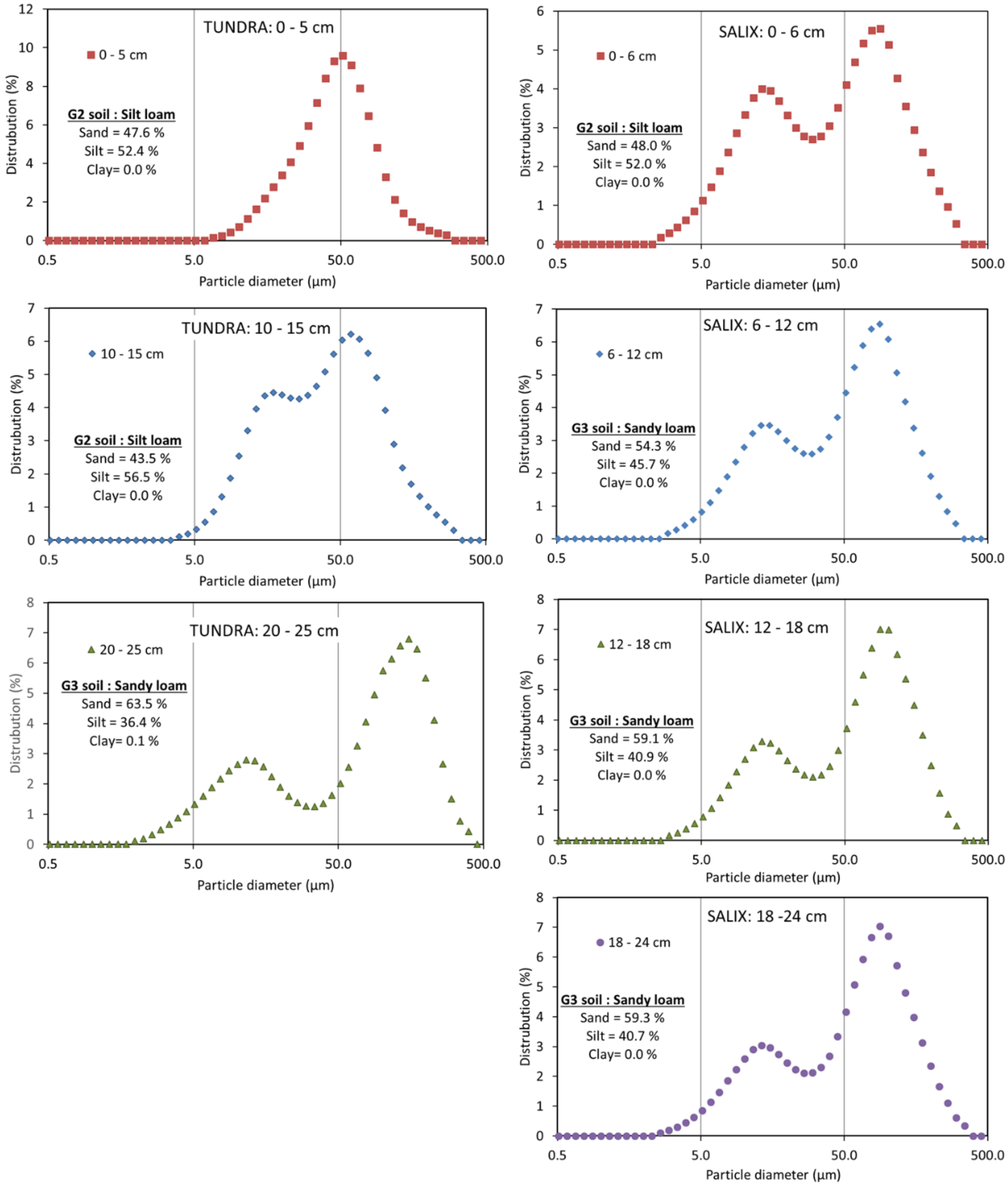

Fig. 2. Granulometric analysis of ground samples. Left: from TUNDRA. Right: from SALIX. Data from several depth ranges are presented. The fractions of sand, silt and clay, based on size distribution, is indicated in each graph. Clay is grains <2 µm, silt is grains between 2 and 50 µm, and sand is grains >50 µm.

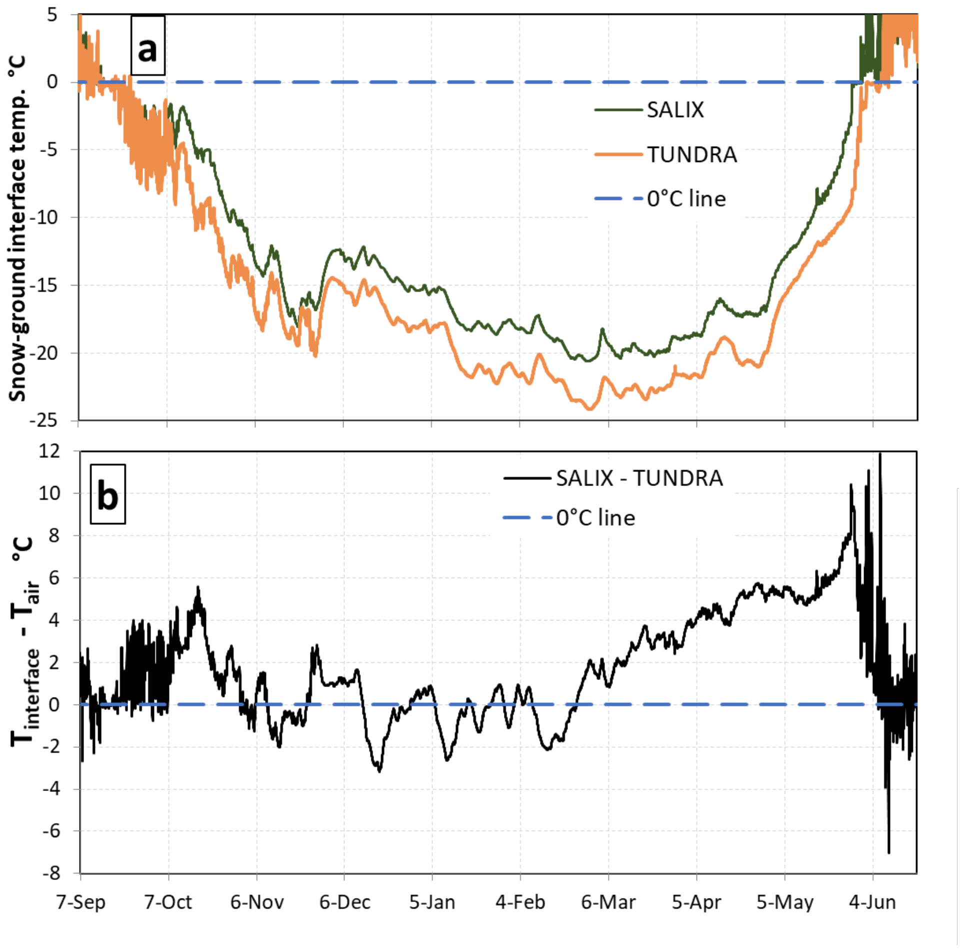

Fig. 3. Temperature data of the snow-ground interface. (a) One-week running mean temperatures at SALIX and TUNDRA. The ground is most of the time warmer at SALIX, but an actual comparison requires correction for differences in air temperature. (b) Temperature difference of the snow-ground interface between SALIX and TUNDRA, corrected for air temperature. This shows that at equal air temperature, from December through March, the ground temperature is most of the time colder at SALIX than at TUNDRA. The dashed horizontal lines are the 0°C lines, added as visual aides.

Ground field measurements

Three ground density profiles were made at or near SALIX. Two of them, dug within 15 m of SALIX, showed values increasing from 1500 to 1850 kg m^{-3} between 5 and 30 cm depth. The third profile, 50 m away, where the shrub density was lower, had values around 1250 kg m^{-3} down to 10 cm depth. These values are fairly similar to those at TUNDRA, where density increased from 1000 kg m^{-3} at 3 cm depth to 1850 kg m^{-3} at 24 cm depth. Regarding ground thermal conductivity, four profiles were measured near SALIX in July 2016 and 2017, yielding values between 0.25 W m^{-1} K^{-1} at 2 cm depth to 1.7 W m^{-1} K^{-1} at 25 cm depth. The profiles measured where the ground temperature sensors were located ranged from 0.3 at 3 cm depth to 1.2 W m^{-1} K^{-1} at 30 cm depth. Around 25 cm depth, values ranged from 0.6 to 1.7 W m^{-1} K^{-1}. Overall, these profiles are fairly similar to those measured at TUNDRA [2]. Granulometric analyses of ground samples were performed and are shown in Fig. 2. Except for the top ground layer where grains around 10 µm are absent at TUNDRA, granulometry is rather similar at both sites. In summary, given variations between nearby spots at each site, there does not appear to be any significant difference in density and thermal conductivity between TUNDRA and SALIX. Except for the top ground layer, granulometry is similar at both sites.

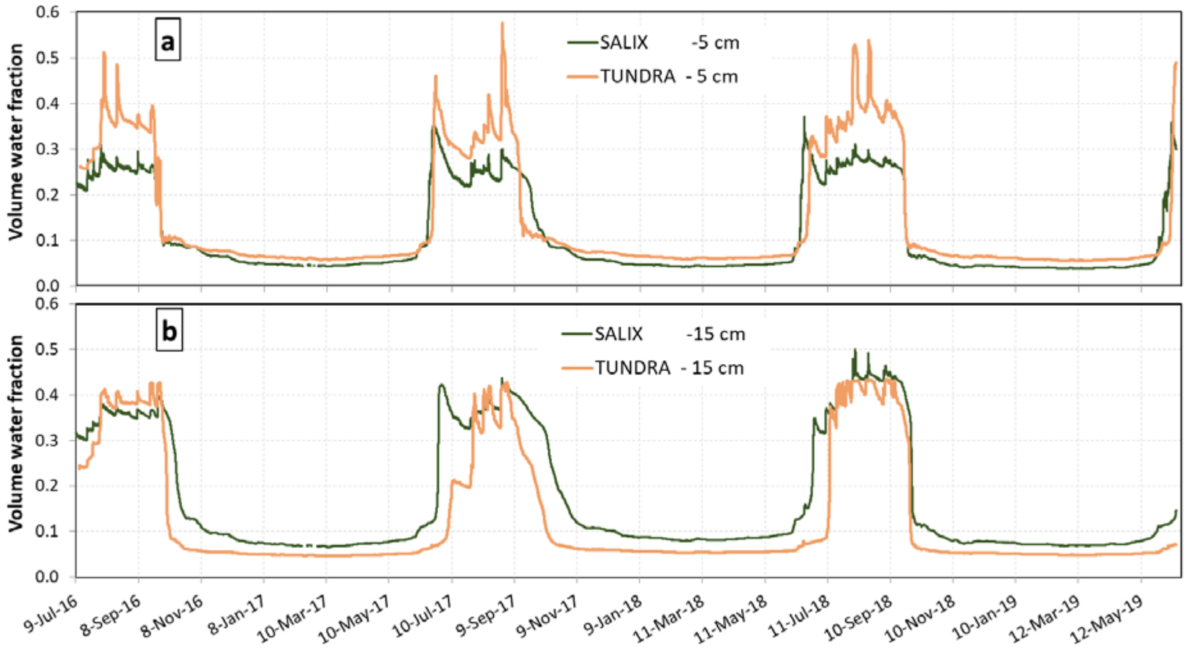

Fig. 4. Ground volume liquid water content at 5 and 15 cm depth at SALIX and TUNDRA for the 3 years studied. In general, the summer water content is greater at TUNDRA at 5 cm depth, but values for SALIX and TUNDRA are similar at 15 cm depth.

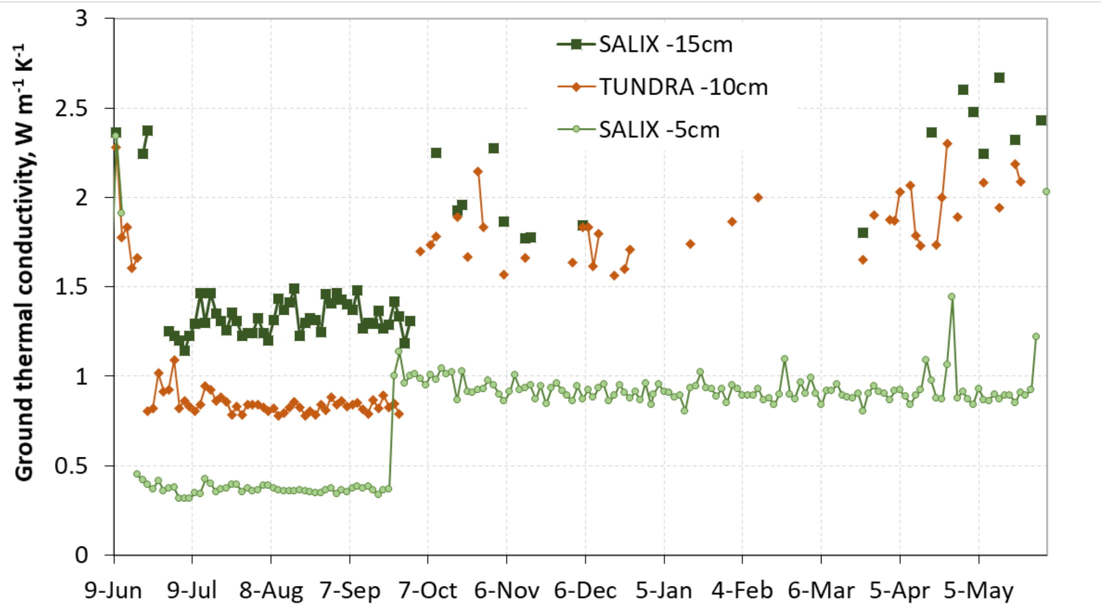

Fig. 5. Ground thermal conductivities at 5 and 15 cm depth at SALIX and at 10 cm depth at TUNDRA. Essentially, these variables only vary with freezing and thawing. For TUNDRA at 10 cm depth and SALIX at 15 cm depth, many values in the frozen state are missing because of insufficient data quality (see Methods), which also results in noisy time series.

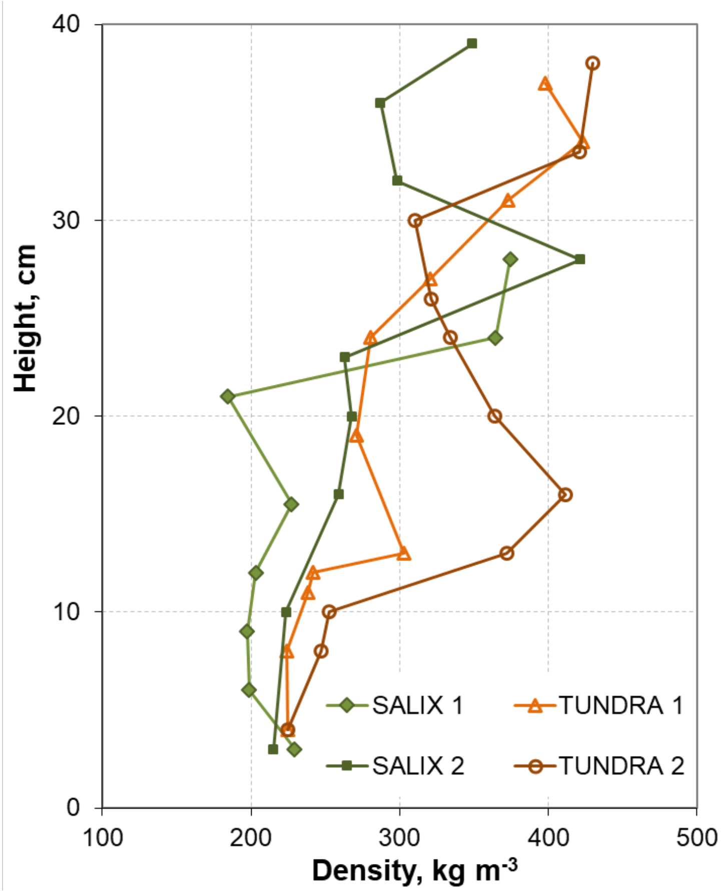

Fig. 6. Snow density profiles measured in pits near the SALIX and TUNDRA sites in mid-May 2019.

Table 1. Values of snow and ground physical variables used for MFM simulations. Values change over time because of ground freezing and snow metamorphism so that different values have been used for several time ranges. Mean values of snow thermal conductivities measured over the periods considered are also indicated.

**Variable**	**Measured k_{snow}, TUNDRA**	**Simulated, TUNDRA**	**Mult. factor[a], k_{snow}**	**Measured k_{snow}, SALIX**	**Simulated, SALIX**	**Mult. factor[a], k_{snow}**
Simulation start Date		15-sept			15-sept	
		**Snow properties**				
ρ_{snow},[b] 0-10 cm		220			210	
ρ_{snow}, 10-20 cm		325			240	
ρ_{snow}, 20-40 cm		350			330	
k_{snow},[c] winter, 0-10 cm	0.07	0.084	1.20	0.07	0.098	1.40
k_{snow}, winter, 10-20 cm	0.07	0.084	1.20	0.04	0.056	1.40
k_{snow}, winter, 20-40 cm	0.23	0.276	1.20	0.09	0.126	1.40
Date ''spring'' period[d]		01-Feb			01-Janv	
k_{snow}, spring, 0-10 cm	0.07	0.084	1.20	0.05	0.085	1.70
k_{snow}, spring, 10-20 cm	0.11	0.132	1.20	0.06	0.102	1.70
k_{snow}, spring, 20-40 cm	0.24	0.288	1.20	0.09	0.153	1.70
Date ''melt'' period[e]		01-May			01-May	
k_{snow}, melt, 0-10 cm	0.18	0.216	1.20	0.06	0.102	1.70
k_{snow}, melt, 10-20 cm	0.14	0.168	1.20	0.07	0.119	1.70
k_{snow}, melt, 20-40 cm	0.30	0.36	1.20	0.14	0.238	1.70
		**Ground properties**				
ρ_{ground},[b] summer, 0-10 cm		1320			1320	
ρ_{ground}, summer, 10-500 cm		1800			1800	
$C_{p,ground}$,[f] summer, 0-10 cm		600000			600000	
$C_{p,ground}$, summer, 10-500 cm		1667			1667	
k_{ground},[c] summer, 0-10 cm		0.5			0.5	
k_{ground}, summer, 10-500 cm		1.2			1.2	
Ground Freezing date[h]		30-oct			26-oct	
ρ_{ground},[b] frozen, 0-10 cm		1320			1320	
ρ_{ground}, frozen, 10-500 cm		1800			1800	
$C_{p,ground}$,[f] frozen, 0-10 cm		1000			1000	
$C_{p,ground}$, frozen, 10-500 cm		1000			1000	
k_{ground},[c] frozen, 0-10 cm		0.8			0.8	
k_{ground}, frozen, 10-500 cm		2			2	

Initial ground temperature, 15 September[i]		
Depth, cm	Temperature	Temperature
30	0	0
5	0	0
0	0	0
-5	0	0
-10	0.5	0
-30	-0.5	0
-100	-2	-4
-200	-6	-7
-500	-11	-11

[a]Factor by which measured thermal conductivity values have been multiplied to account for the artifact caused by the heated needle probe method. For SALIX, the factor has been enhanced to attempt to improve the fit between data and simulations, thus evidencing that a process other than conduction through the snow is taking place.

[b]Density, kg m^{-3}

[c]Thermal conductivity, W m^{-1} K^{-1}

[d]Different time periods are used to account for time-changes in snow thermal conductivity. “spring” is a time period and does not necessarily relate to a season

[e]Same as [c]. “melt” indicates snow metamorphism in spring, when the temperature gradient reverses, and does not necessarily imply that snow actually started melting on May 1st

[f]Specific heat capacity, J kg^{-1} K^{-1}

[g]This very high value is to simulate ground freezing, during which ground temperature does not change. The model does not actually simulate the phase changes of water.

[h]Date when “frozen”parameter values start being used. This is not the actual observed ground freezing data as this is depth-dependent. The date is chosen to optimize the agreement between simulations and data.

[i]Based in part on measurements in the active layer at the sites. At depth, values are infered from thermistors in other parts of the valley.

References

1 Gauthier, G. *et al.* The tundra food web of Bylot Island in a changing climate and the role of exchanges between ecosystems. *Ecoscience* **18**, 223-235, doi:10.2980/18-3-3453 (2011).

2 Domine, F., Lackner, G., Sarrazin, D., Poirier, M. & Belke-Brea, M. Meteorological, snow and soil data (2013–2019) from a herb tundra permafrost site at Bylot Island, Canadian high Arctic, for driving and testing snow and land surface models. *Earth Syst. Sci. Data Discuss.* **2021**, 1-23, doi:10.5194/essd-2021-54 (2021).
